# Effect of drying system, layer thickness and drying temperature on the drying parameters, product quality, energy consumption and cost of the marjoram leaves

**DOI:** 10.1038/s41598-024-55007-7

**Published:** 2024-02-26

**Authors:** El-Sayed G. Khater, Adel H. Bahnasawy, Mai H. Abd El-All, Hassan M. M. Mustafa, Ahmed M. Mousa

**Affiliations:** 1 Agricultural and Biosystems Engineering Department, Faculty of Agriculture, Benha University, Moshtohor, P.O. Box 13736, Toukh, Kalubia Egypt; 2 Mechanical Engineering Department, Engineering and Renewable Energy Research Institute, National Research Centre, Giza, Egypt; 3 Agricultural Machinery and Power Engineering Department, Faculty of Agricultural Engineering, Al-Azhar University, Cairo, Egypt

**Keywords:** Drying rate, Essential oil content, Energy, Cost, Marjoram, Solar drying, Ecology, Environmental sciences, Engineering

## Abstract

The main aim of this work is to study the main factors affecting the quality of the dried product and the energy requirements through optimizing these factors. To achieve that different drying systems (solar, hybrid solar and oven dryings), layers thickness (1, 2 and 3 cm) and drying temperatures (50, 60 and 70 °C) were used. The obtained results indicated that, the accumulated weight loss of marjoram leaves ranged from 73.22 to 76.9%, for all treatments under study. The moisture content of marjoram leaves ranged from 273.39 to 333.17, 258.02 to 333.04 and 269.38 to 324.90% d.b. for hybrid solar, oven and solar drying systems, respectively. The highest value of the drying rate of marjoram leaves (223.73 g_water_ kg^−1^ h^−1^) was obtained when the marjoram dried by oven drying system at 70 °C at 1 cm layer thickness. The highest values of the basil and marjoram oil content (2.91%) was obtained when the marjoram dried under sun drying system. The energy consumption for drying marjoram decreases with increasing drying temperature and layer thickness for hybrid solar and oven drying systems. The cost of dried marjoram dried under hybrid solar drying system was lower than those of oven drying system, the highest cost (13.48 LE kg^−1^) was obtained at a temperature of 50 °C and a layer thickness of 1 cm.

## Introduction

The need for food production in sustainable and stable way became big challenge for the whole world. Controlling the population growth and increasing the food and agricultural production is the proper solution the rapid growth of population result in high consumption of non-renewable energy. Famine is the major challenge according to the Food and Agriculture Organization (FAO), food waste management could affect the famine problem by 1.3 billion tons, this means that, the global manufactured nutrition is squandered by 30% yearly^1,2^.

There is an increasing attention in natural antioxidants found in plants because of the world-wide trend toward the use of natural additives in food and cosmetics. Herbs and spices are one of the most important targets to search for natural antioxidants from the point of view of safety. the antioxidative effects of rosemary, ginger, thyme, oregano, sage, marjoram, red pepper, black pepper, peppermint, clove, cinnamon, summer savory, coriander, common balm, basil, spearmint, nutmeg, fennel, parsley, garlic, cumin, etc. Among the herbs of the Labiatae family^3^.

Spices and herbs are considered necessary component in the daily food consumed by the human and present an essential role in preserving food, curing illness, and enhancing cosmetics. Suitable processing is required because of its high moisture content and often high load of microorganisms. Drying is the most common way used to decrease moisture content and hence the water activity to a safe limit which prolongs shelf life^4^. However, consumers’ demand on processed products with most of the original characteristics of the fresh plants has increased. Consequently, drying must be done carefully to keep the taste, aroma, color, appearance, as well as nutritional value of the plants to maximum possible extent. Besides to quality considerations, drying efficiency is another key aspect for evaluating drying performance^5,6^.

Energy is most important to human existence, its sources of generation, methods of utilization, impacts on the environment and its economic importance are a high concern to every nation. Because of that, scientists are working hard and with great care and precautions to devise techniques to make sure all energy projects are environmentally friendly, economically affordable, and efficiently utilizable as well as sustainable in nature before implementation. Solar energy would be the best solution to complement the interest of the global community to relieve the radically changing demand for pure and sustainable energy^7,8^. Motevali and Minael^9^ studied the effect of microwave pre-treatment, drying temperature and air velocity on the energy requirements in drying pomegranate arils. They concluded that the energy utilization and drying time decreased considerably with microwave pre-treatment of pomegranate arils. The minimum values of exergy loss and exergy efficiency were associated with the 200 W microwave pre-treatment, while they were maximum for control treatment. Motevali et al.^10^ studied the effect of different drying methods (hot air, microwave, vacuum, infrared, microwave-vacuum and hot air-infrared) on the energy consumption in drying mushroom. They concluded that using microwave recorded the lowest energy consumption level compared to using vacuum dryers consumed the highest energy in drying. Using hot air in drying showed reduction in energy consumption with increasing temperature. Using hot air-infrared decreased energy consumption relative to infrared only.

Drying adds many benefits to food products, like, weight and volume reduction, to transportation costs lowering and product shelf life extensions without refrigeration. Furthermore, it produces intermediate or convenience food products. Drying consists of complex mechanisms, such as physical, chemical, and biochemical reactions, making it an unsteady, extremely non-linear, and dynamic, thermal process^11^. However, drying may also lead to undesirable quality changes^12^, such as altering food quality parameters, such as colour^13–17^, shrinkage^18^, rehydration ratio^19^, antioxidant activities^20,21^, and total phenolic compound^13,14^ to name but a few.

The influence of drying on the volatile content of several aromatic plants and vegetables has been widely studied. These studies showed that factors, such as the drying method, time, and temperature, clearly affected the concentrations of volatile compounds during the drying process. In this sense, the scientific literature is scarce or even non-existing, under our knowledge, regarding marjoram drying and the composition of its essential oil^22^. Marjoram is one of the most worldwide used herbs in Egypt due to its antioxidant, anti-inflammatory, anti-genotoxic, antimutagenic, anticoagulant, and beneficial effects^23^. The members of the family Labiatae are most commonly used for culinary purposes, based on their characteristic aroma and flavours. They are generally grown in the Mediterranean region, especially in Egypt. Marjoram (*Origanum majorana* L., syn. Majorana hortensis Moench) is considered one of the most common herbs of this family and is native to Southern Europe and the Mediterranean region, especially Egypt^24^.

Optimizing the factors affecting the performance and quality of the dried products is very important which in turn affects the energy requirement and drying cost. Solar drying is not useful at night, has low efficiency, occasionally overheats, changes in quality, and cannot accurately extract the sun’s energy. In some circumstances, such as overcast and wet regions, it is challenging to gather solar energy; solar collector drying efficiency is very poor; the environment is contaminated; and there are excessive energy losses, all of which have an impact on the product’s quality while drying. Hybrid solar drying is a suitable alternative way to overcome all of these defects, while maintaining the quality of herbal plants and speeding up the drying process. Also, temperature of drying and plant layer thickness are the most important factors affecting the product quality and shelf life after drying, therefore, the main aim of this work is to study the effect of drying system, drying temperature and layer thickness on the drying parameters of marjoram, quality product, energy consumption and drying cost under hybrid solar drying system.

## Materials and methods

The experiment was carried out at Agricultural and Bio-Systems Engineering Department, Faculty of Agriculture Moshtohor, Benha University, Egypt (latitude 30° 21′ N and 31° 13′ E). During the season 2022 from May to August under the regulations of International, National and Benha University which are consistent with the national and international guidelines and legislation. The ambient air temperature ranged from 27.3 to 36.8 °C, the relative humidity ranged from 38.1 to 73.5% and solar radiation ranged from 338.2 to 971.9 kJ m^−2^ day^−1^.

### Materials

The fresh marjoram (*Origanum majorana* L.) was harvested from the Experimental Research Station, Faculty of Agriculture Farm, Moshtohor, Benha University at moisture content ranged from 77 to 87%. W.b.

#### Drying systems

The marjoram was dried using two systems as follows:

##### Hybrid solar drying

**Figure 1:**
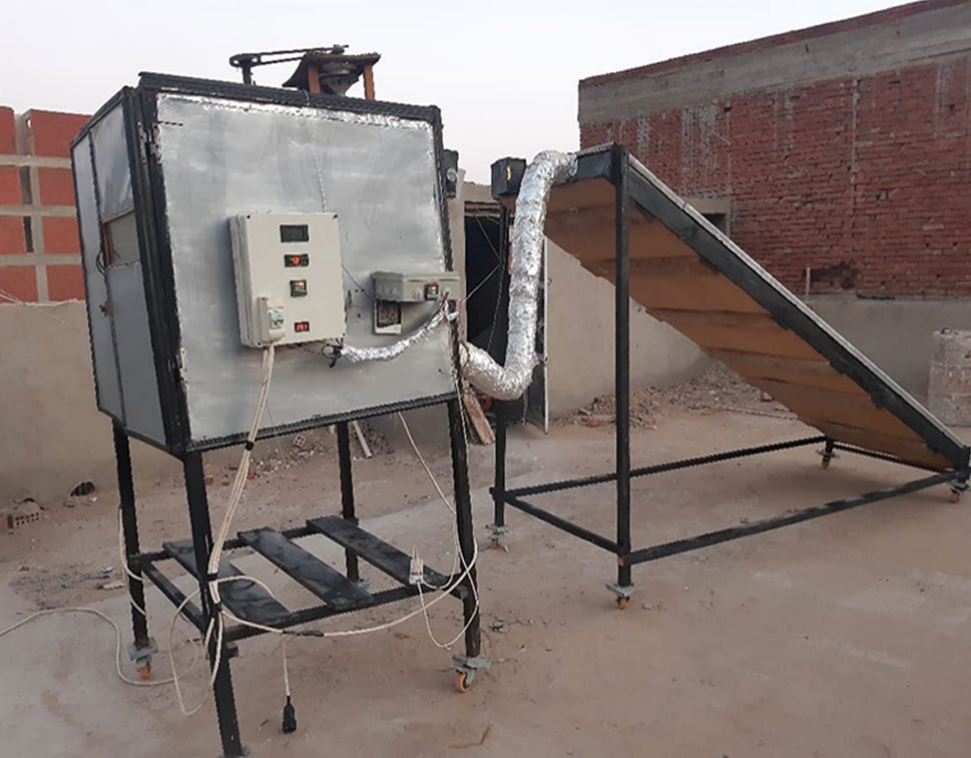
Hybrid solar dryer.

Figure 1 illustrates the hybrid solar drying system description. It shows the system which consists of solar collector, drying chamber, trays, fans, electric heater and control unit.

- The solar collector

**Figure 2:**
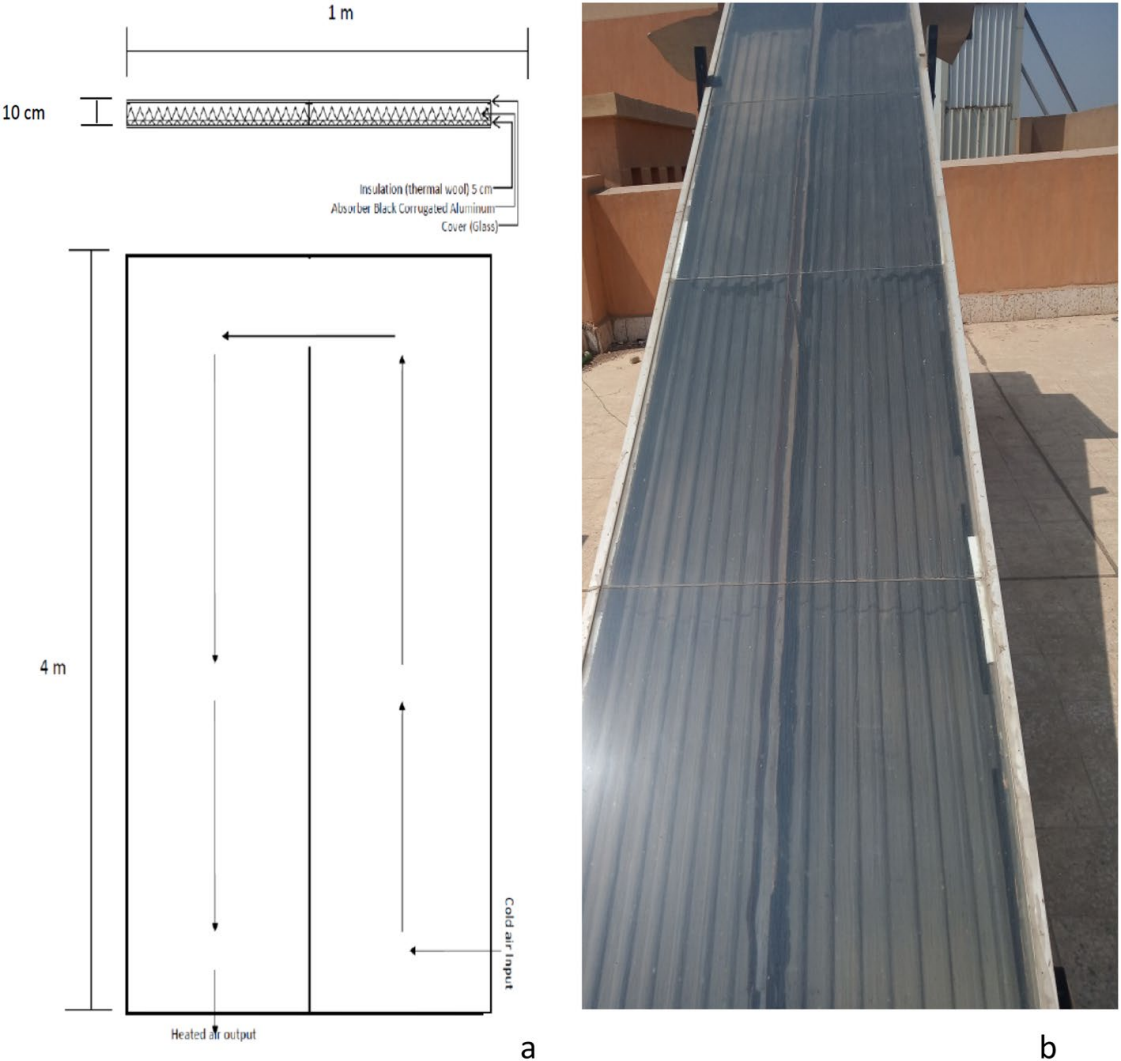
Solar collector. (**a**) Geometric view (**b**) Top view.

The solar collector consists of three major components, namely: The glass cover has dimensions of 4.0 m length, 1.0 m wide and 5.0 mm thickness. The cover is fixed on a wooden frame with a thickness of 10 cm. It is divided into two lines, 50 cm wide each. The absorber plate is made from corrugated black aluminum plate. The insulation is a thermal wool with a 5.0 cm thickness as shown in Fig. 2.

- The drying chamber

**Figure 3:**
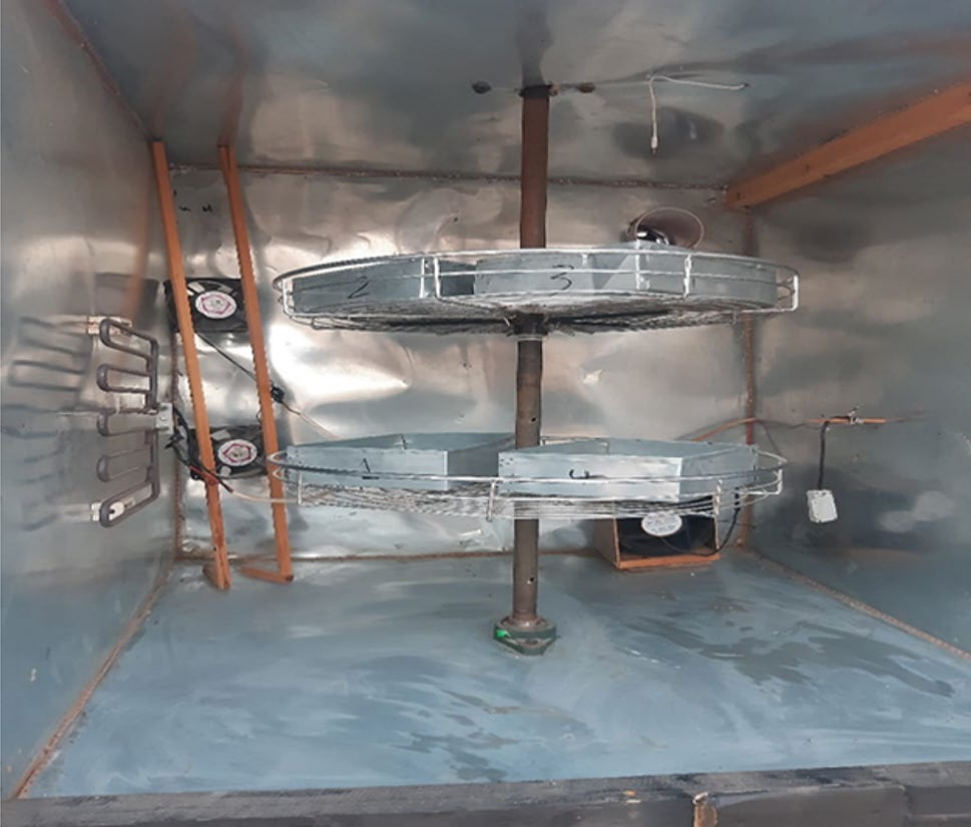
The drying chamber.

The drying chamber has a length of 1.0 m, width of 1.0 m and height of 1.0 m. It is made of galvanized steel (5 mm thickness). The drying chamber is loaded on four ivory wheels for easy movement with the collector. The distance between the inner and outer surface of the drying chamber is 5 cm. Insulating materials (thermal wool with a thickness of 2 cm and a layer of foam with a thickness of 3 cm) have been placed to reduce heat dissipation from the walls as shown in Fig. 3.

- The trays

The trays are made of stainless steel and have a length of 0.30 m, width of 0.20 m and height of 0.07 m. They have perforated bottom which allows heated air to pass through products.

- The blower

Two air blowers were used to force and re-circulate the drying air to the drying chamber (Model C.C.P. Parma—Flow Rate 6.6 m^3^ h^−1^—RPM 2800—Power 150 W, 220 V 50 Hz, Italy).

- Temperature control unit

Digital temperature controller (REX_C100 PID) it is used to control the start and stop of the additional heat source (the electric heater 2000 watts) to ensure the stability of the temperature inside the drying room.

- Electric heater

An electric heater with a capacity of 2000 watts was used to heat the air inside the drying room as an additional energy source with solar energy.

- Control units

**Figure 4:**
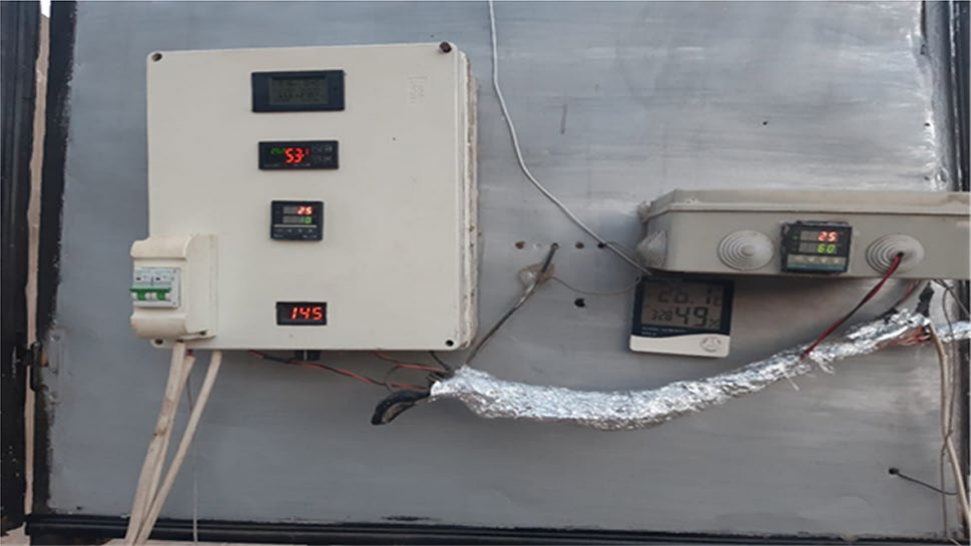
Image of control units.

Controllers are used to control the air temperature and air humidity inside the drying room. It consists of two control units. The first consists of a device for measuring electrical energy consumption, a device for measuring temperature, a device for measuring humidity, a temperature sensor, a humidity sensor inside the drying room, and a device for controlling the speed of fans. The first control unit is used to control the air temperature and humidity inside the drying room, in addition to measuring the electrical energy required for the drying process, as well as controlling the speed of fans and trolleys. The second control unit consists of a temperature control device and a temperature sensor to control the operation and disconnection of the fan for drawing hot air from the solar collector into the drying room. Figure 4 shows the control units.

- Trolley rotation control unit

**Figure 5:**
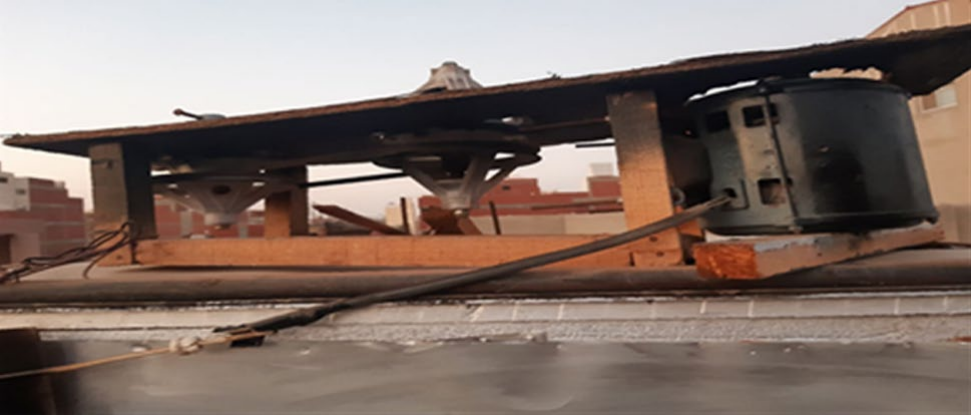
Trolley rotation control unit.

The trolley rotation control unit consists of a motor that rotates the trolley inside the drying room at different speeds, and 2 gear boxes that reduce the motor speed to reach the required speeds as shown in Fig. 5.

##### Oven drying

The solar dryer was used in setting ovens using the electric heater only after separating the solar collector by separating the intake fan, and a gate was used to prevent hot air from entering the room from the solar collector. The drying chamber has a length of 1.0 m, width of 1.0 m and height of 1.0 m, and the distance between the inner and outer wall of the room was 5 cm. Insulating materials were placed in this space (thermal wool with a thickness of 2 cm, and a cork board with a thickness of 3 cm) to prevent heat leakage from inside the room as shown in Fig. 3.

### Methods

Marjoram was cleaned by removing undesired stems and waste materials.

#### Treatments

In this study, the treatments include: two drying systems (hybrid solar and oven systems), drying temperatures (50, 60 and 70 °C) and three layers thickness were (1, 2 and 3 cm). The experimental design was a split plot design with three replicates.

#### Measurements and determination

- Drying measurements

The mass was measured by electric digital balance (Model HG—5000—Range 0–5000 g ± 0.01 g, Japan) hourly for hybrid solar and oven drying methods. The electrical energy needed for each drying process was measured using a device Digital multimeter for voltage and current (PZEM-061). The temperature inside the drying chamber was controlled using a device (Model C100FK02-M*AN, Range 0–1200 °C). The air humidity inside the drying chamber was controlled using a device (Model OKE-2020H, Range 0–100%). The speed of the propellers and the rotation of the trolley were controlled using a device DIMR Voltage Regulator. Temperature and relative humidity of outside air were recorded by using a Perfect prime Data Logger (Model TH165—Range − 20 to 70 °C and 5–95% RH, Australia) every hour.

##### Moisture content

Moisture content of the fresh and dried marjoram leaves were determined using conventional laboratory oven kept at 105 °C until constant weight was reached according to ASAE standard^25^. Triplicate determinations were made, and the moisture content calculated as the following equation:

1 $$M{\text{C}} = \frac{{{\text{M}}_{{{\text{wet}}}} - M_{dry} }}{{{\text{M}}_{{{\text{dry}}}} }} \times {100}$$

where: MC is the moisture content, (% d.b.)

M_wet_ is the wet mass of samples, (g).

M_dry_ is the dry mass of samples, (g).

##### Drying rate

The drying rate (DR) of marjoram was calculated using the following equation according to Fadhel et al.^26^:

2 $$DR = \frac{{{\text{M}}_{{{\text{t}} + {\text{dt}}}} - M_{t} }}{{{\text{dt}}}}$$

where: DR is the drying rate, (kg_water_/kg_dry base_.hr).

M_t_ is the moisture content at any time t, (% d.b.)

M_t+dt_ is the moisture content at t + dt, (% d.b.).

t is the drying time, (h).

#### The quality of dried product

##### Extraction of essential oil

Spearmint essential oil was extracted using steam distillation method from fresh and dried spearmint leaves for 3 h as described by Mkaddem et al.^27^. The essential oils were dried over anhydrous sodium sulfate and stored at − 18 °C, till analysis.

##### GC/MS analyses

Analysis of the samples were conducted by using a gas chromatography (Agilent 8890 GC System), coupled to a mass spectrometer (Agilent 5977B GC/MSD) and equipped with a HP-5MS fused silica capillary column (30 m, 0.25 mm i.d., 0.25 mm film thickness). The oven temperature was maintained initially at 50 °C, then programmed from 50 to 200 °C at a rate of 5 °C min^−1^ and from 200 to 280 °C at a rate of 10 °C min^−1^, then held for 7 min at 280 °C. Helium was used as the carrier gas, at flow rate of 1.0 mL min^−1^. The essential oil was dissolved in diethyl ether (20 µL essential oil/mL diethyl ether), and then 1 µL of this solution were injected in the GC with a split ratio 1:50. The temperature of injection was 230 °C. Mass spectra in the electron impact mode (EI) were obtained at 70 eV and scan m/z range from 39 to 500 amu. The isolated peaks were identified by matching them with data from the library of mass spectra (National Institute of Standard and Technology, NIST). The obtained chromatogram and report of GC analysis for each sample were analyzed to calculate the percentage of main components of volatile oil^28^.

#### Power and energy requirement

The power requirement (kW) was estimated by using the clamp meter to measure the line current strength (I) and the potential difference value (V).

The total electric power requirement under machine working load (P) was calculated according to Kurt^29^ by the following equation:

3 $$P = \frac{I \times V \times \cos \theta }{{{1000}}}$$

where: P is total electric power, (KW).

I is the electric current, (Amperes).

V is the electrical voltage, (volt).

cosθ is the power factor being equal to 0.85.

### Cost calculation for the drying marjoram

The costs were calculated according to Khater et al.^30^. Table 1 shows inputs of drying cost components for dryer operate for marjoram drying.

**Table 1:** Inputs of costs calculations.

Items	Cost, LE
Price of equipment, LE	15,000
Motor, kW	1.0
Life expected, year	5
Taxes, %	3
Repair, %	10
Interest, %	12
Labors, LE h^−1^	20

### Statistical analysis

The data were subjected to analysis using statistical package SPSS version 21 in which one way ANOVA and Duncan Multiple Range Test (DMRT) were performed at significance level of (p < 0.05) at 95% confidence limit to know the significant differences between the treatment means for different parameters according to Duncan^31^.

## Results and discussion

### Accumulated weight loss of marjoram leaves

**Figure 6:**
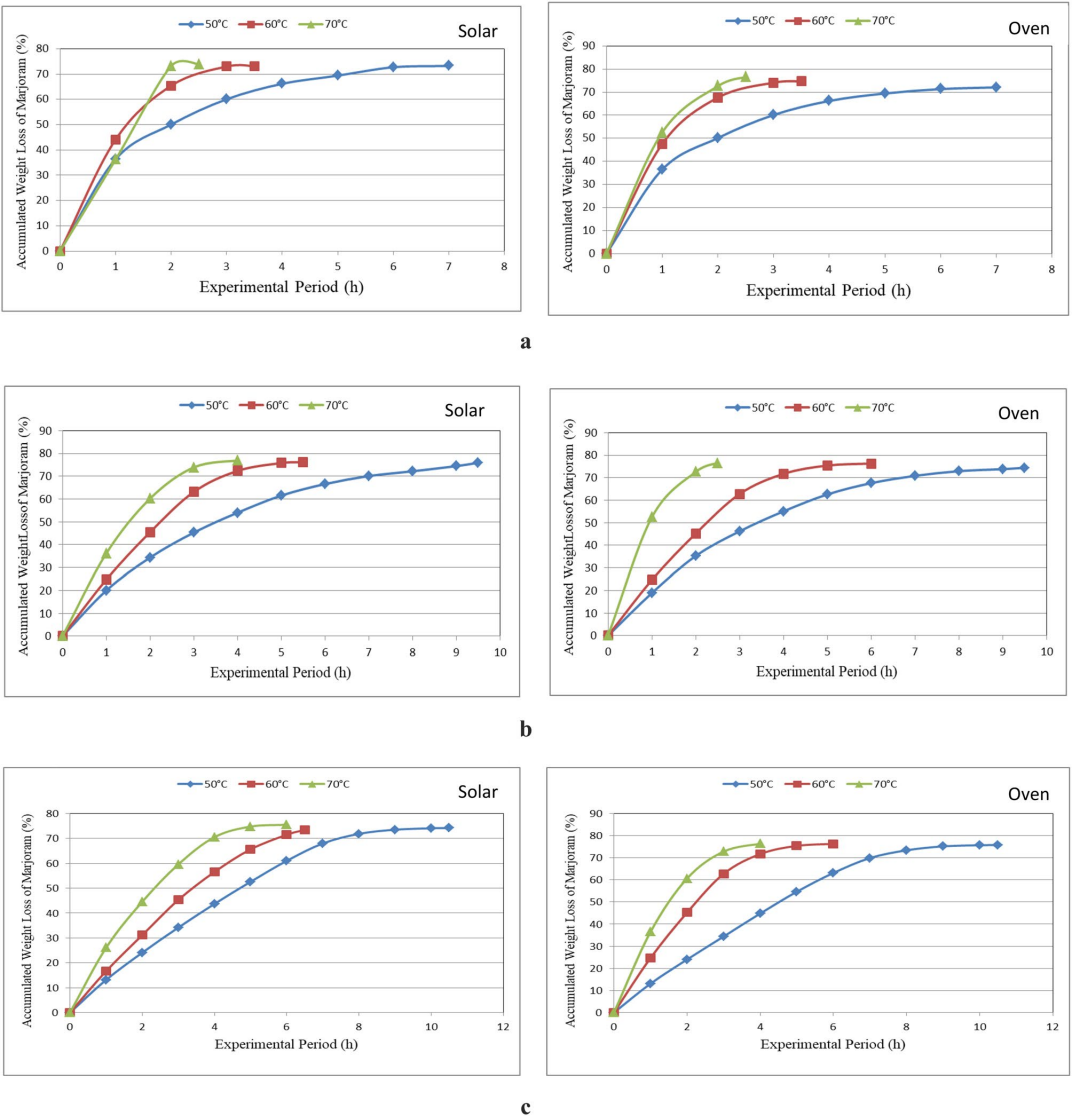
(**a**) The accumulated weight loss of marjoram leaves at 1 cm thickness layer for different drying systems. (**b**) The accumulated weight loss of marjoram leaves at 2 cm thickness layer for different drying systems. (**c**) The accumulated weight loss of marjoram leaves at 3 cm thickness layer for different drying systems.

Figure 6a–c show the accumulated weight loss of marjoram leaves that dried in different drying systems (hybrid and oven drying) under different layers thicknesses (1, 2 and 3 cm) and different drying temperatures (50, 60 and 70 °C) during drying period. The results indicate that the accumulated weight loss of marjoram leaves increases with increasing layer thickness and drying temperature during drying period. It could be seen that the accumulated weight loss of marjoram leaves significantly increased from 36.34 to 73.22, 44.08 to 73.07 and 53.54 to 73.81%, when the drying period increased from 1 to 7, 1 to 3.5 and 1 to 2.5 h, respectively, for 50, 60 and 70 °C drying temperature at 1 cm thickness layer for hybrid solar drying system. For oven drying system, the accumulated weight loss of marjoram leaves increased from 36.41 to 72.05, 47.49 to 74.64 and 52.5 to 76.53%, when the drying period increased from 1 to 7, 1 to 3.5 and 1 to 2.5 h, respectively, for 50, 60 and 70 °C drying temperature at 1 cm layer thickness compared to increased from 32.27 to 76.04%, when the drying period increased from 1 to 10 h, respectively, for 1 cm layer thickness for solar drying system. The trend of these results agreed with those obtained by Seyfi et al.^32^ and Khater et al.^7^ whose found the accumulated weight loss increases with increasing layer thickness and drying temperature during drying period.

For 2 cm layer thickness, the accumulated weight loss of marjoram leaves significantly increased from 19.98 to 75.9, 24.72 to 76.14 and 36.17 to 76.90%, when the drying period increased from 1 to 9.5, 1 to 5.5 and 1 to 4.0 h, respectively, for 50, 60 and 70 °C drying temperature for hybrid solar drying system. For oven drying system, the accumulated weight loss of marjoram leaves significantly increased from 18.89 to 74.41, 24.68 to 76.28 and 36.46 to 76.35%, when the drying period increased from 1 to 9.5, 1 to 5.0 and 1 to 4.0 h, respectively, for 50, 60 and 70 °C drying temperature compared to increased from 36.61 to 73.04%, when the drying period increased from 1 to 11 h, respectively, for 2 cm thickness layer for solar drying system.

For 3 cm layer thickness, the accumulated weight loss of marjoram leaves significantly increased from 13.16 to 74.19, 16.66 to 73.45 and 26.07 to 75.51%, when the drying period increased from 1 to 10.5, 1 to 7.5 and 1 to 6.0 h, respectively, for 50, 60 and 70 °C drying temperature for hybrid solar drying system. For oven drying system, the accumulated weight loss of marjoram leaves significantly increased from 13.07 to 75.77, 16.5 to 74.71 and 25.93 to 76.90%, when the drying period increased from 1 to 10.5, 1 to 7.5 and 1 to 6.0 h, respectively, for 50, 60 and 70 °C drying temperature compared to increased from 36.61 to 73.04%, when the drying period increased from 1 to 11 h, respectively, for 2 cm layer thickness for solar drying system.

The results indicate that the accumulated weight loss of marjoram leaves increases with increasing drying temperature for hybrid solar and oven drying systems, it could be seen that the accumulated weight loss of marjoram leaves increased from 73.22 to 73.81, 75.9 to 76.9 and 74.19 to 75.51% when the drying temperature increased from 50 to 70 °C for 1, 2 and 3 cm thickness layer, respectively, for hybrid solar drying system. Also, the accumulated weight loss of marjoram leaves increased from 72.05 to 76.53, 74.41, 76.36 and 75.77 to 76.9% when the drying temperature increased from 50 to 70 °C for 1, 2 and 3 cm layer thickness, respectively, for oven drying system.

The results also indicate that the shorter drying period (2.5 h) was occurred under the 1 cm layer thickness at drying temperature of 70 °C. Meanwhile, the longer drying period (10.5 h) was occurred for the 3 cm layer thickness at drying temperature of 50 °C for hybrid solar and oven drying systems compared to the longer drying period was 13 h for solar drying system. The trend of these results agreed with those obtained by Khater and Bahnasawy^7^ and Khater et al.^33^.

### Moisture content of marjoram leaves

**Figure 7:**
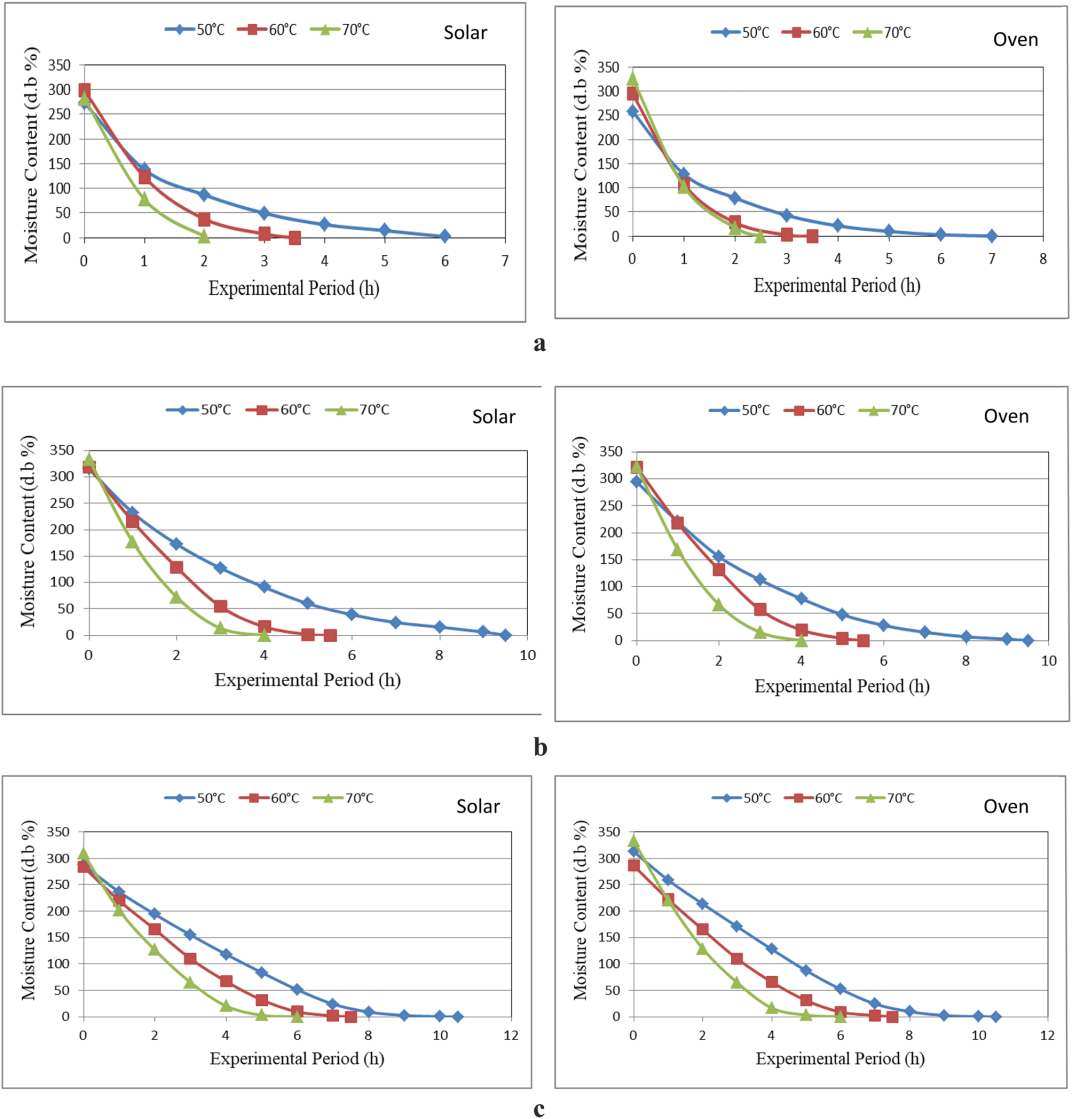
(**a**) Moisture content of marjoram Leaves at 3 cm thickness layer for different drying systems. (**b**) Moisture content of marjoram Leaves at 3 cm thickness layer for different drying systems. (**c**) Moisture content of marjoram Leaves at 3 cm thickness layer for different drying systems.

Figure 7a–c show the moisture content of marjoram leaves that dried in different drying systems (hybrid solar and oven drying) under different layers thicknesses (1, 2 and 3 cm) and different drying temperatures (50, 60 and 70 °C) during drying period. The results indicate that the moisture content of marjoram leaves decreases with increasing layer thickness and drying temperature during experimental period. It could be seen that the moisture content of marjoram leaves significantly decreased from 273.39 to 1.93, 298.10 to 0.05 and 282.11 to 2.42% d.b., when the drying period increased from 1 to 7.0, 1 to 3.5 and 1 to 2.0 h, respectively, for 50, 60 and 70 °C drying temperature at 1 cm layer thickness for hybrid solar drying system. For oven drying system, the moisture content of marjoram leaves significantly decreased from 258.02 to 0.047, 294.18 to 0.052 and 326.10 to 0.056% d.b., when the drying period increased from 1 to 7.0, 1 to 3.5 and 1 to 2.5 h, respectively, for 50, 60 and 70 °C drying temperature at 1 cm layer thickness compared to decreased from 324.90 to 1.80%, when the drying period increased from 1 to 9 h, respectively, for 1 cm layer thickness for solar drying system.

For 2 cm layer thickness, the moisture content of marjoram leaves significantly decreased from 315.14 to 0.03, 319.04 to 0.03 and 333.17 to 0.028% d.b., when the drying period increased from 1 to 9.5, 1 to 5.5 and 1 to 4.0 h, respectively, for 50, 60 and 70 °C drying temperature for hybrid solar drying system. For oven drying system, the moisture content of marjoram leaves significantly decreased from 294.76 to 0.026, 321.74 to 0.028 and 322.94 to 0.020% d.b., when the drying period increased from 1 to 9.5, 1 to 5.5 and 1 to 4.0 h, respectively, for 50, 60 and 70 °C drying temperature compared to decreased from 270.97 to 1.32%, when the drying period increased from 1 to 11 h, respectively, for 2 cm layer thickness for solar drying system.

For 3 cm layer thickness, the moisture content of marjoram leaves significantly decreased from 287.53 to 0.017, 283.95 to 0.02 and 308.48 to 0.02% d.b., when the drying period increased from 1 to 10.5, 1 to 7.5 and 1 to 6.0 h, respectively, for 50, 60 and 70 °C drying temperature for hybrid solar drying system. For oven drying system, the moisture content of marjoram leaves significantly decreased from 312.81 to 0.018, 286.21 to 0.017 and 333.04 to 0.019% d.b., when the drying period increased from 1 to 10.5, 1 to 7.5 and 1 to 5.5 h, respectively, for 50, 60 and 70 °C drying temperature compared to decreased from 269.38 to 0.02%, when the drying period increased from 1 to 13 h, respectively, for 3 cm layer thickness for solar drying system. The trend of these results agreed with those obtained by Rana et al.^34^ whose found the moisture content increases with increasing layer thickness and drying temperature during drying period.

The results indicate that the moisture content of marjoram leaves increases with increasing drying temperature for hybrid solar and oven drying systems, it could be seen that the moisture content of marjoram leaves increased from 273.39 to 282.11, 315.14 to 333.17 and 287.53 to 308.48% d.b., when the drying temperature increased from 50 to 70 °C for 1, 2 and 3 cm layer thickness, respectively, for hybrid solar drying system. Also, the moisture content of marjoram leaves increased from 258.02 to 326.1, 294.76 to 322.94 and 312.81 to 333.04% d.b., when the drying temperature increased from 50 to 70 °C for 1, 2 and 3 cm layer thickness, respectively, for oven drying system. Increased drying temperature and air recirculating further decrease the relative humidity of a product. This can be explained by the fact that increased temperature and hot airflow inside the drying chamber increases mass and heat transfer, leading to sharper drops in moisture content. The trend of these results agreed with those obtained by Doymaz^35^ and Abd EL-All et al.^36^.

### Drying rate of marjoram leaves

**Figure 8:**
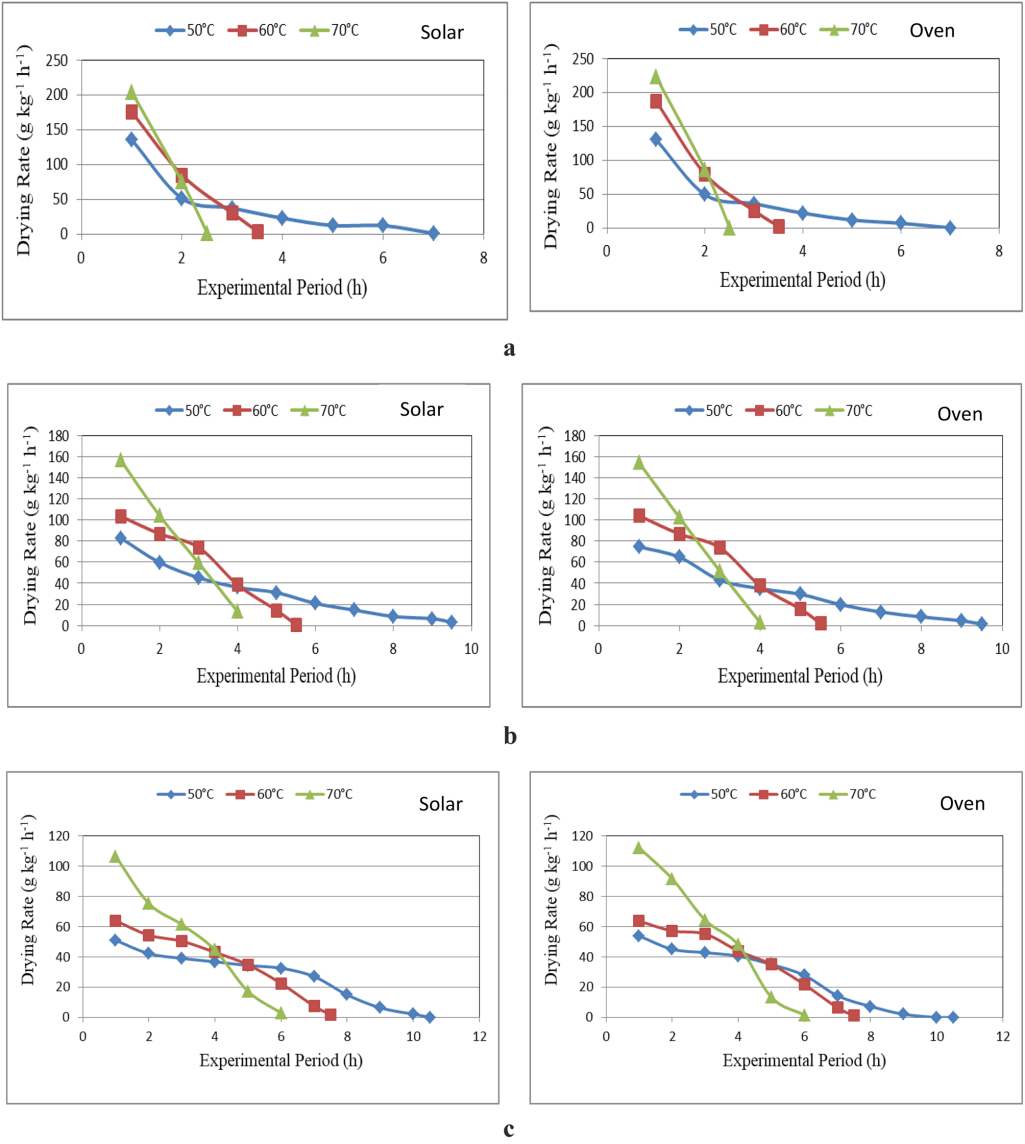
(**a**) Drying rate of marjoram leaves at 3 cm thickness layer for different drying systems. (**b**) Drying rate of marjoram leaves at 3 cm thickness layer for different drying systems. (**c**) Drying rate of marjoram leaves at 3 cm thickness layer for different drying systems.

Figure 8a–c show the drying rate of marjoram leaves that dried in different drying systems (hybrid solar and oven drying) under different layers thicknesses (1, 2 and 3 cm)and different drying temperatures (50, 60 and 70 °C) during drying period. The results indicate that the drying rate of marjoram leaves decreases with increasing layer thickness and drying temperature during experimental period. It could be seen that the drying rate of marjoram leaves significantly decreased from 135.70 to 0.96, 175.55 to 3.69 and 204.60 to 1.21 g_water_ kg^−1^ h^−1^, when the drying period increased from 1 to 7.0, 1 to 4.0 and 1 to 2.5 h, respectively, for 50, 60 and 70 °C drying temperature at 1 cm layer thickness for hybrid solar drying system. For oven drying system, the drying rate of marjoram leaves significantly decreased from 130.36 to 0.023, 187.22 to 2.14 and 223.73 to 0.056 g_water_ kg^−1^ h^−1^, when the drying period increased from 1 to 7.0, 1 to 3.5 and 1 to 2.5 h, respectively, for 50, 60 and 70 °C drying temperature at 1 cm layer thickness compared to decreased from 137.13 to 0.90%, when the drying period increased from 1 to 9.5 h, respectively, for 1 cm layer thickness for solar drying system.

For 2 cm layer thickness, the drying rate of marjoram leaves significantly decreased from 82.95 to 3.00, 103.60 to 0.74 and 156.75 to 0.56 g_water_ kg^−1^ h^−1^, when the drying period increased from 1 to 9.5, 1 to 5.5 and 1 to 4.0 h, respectively, for 50, 60 and 70 °C drying temperature for hybrid solar drying system. For oven drying system, the drying rate of marjoram leaves significantly decreased from 74.60 to 1.17, 104.07 to 1.85 and 154.21 to 2.75 g_water_ kg^−1^ h^−1^, when the drying period increased from 1 to 9.5, 1 to 5.5 and 1 to 4.0 h, respectively, for 50, 60 and 70 °C drying temperature compared to decreased from 113.55 to 3.30%, when the drying period increased from 1 to 11 h, respectively, for 2 cm layer thickness for solar drying system.

For 3 cm layer thickness, the drying rate of marjoram leaves significantly decreased from 51.04 to 0.17, 63.97 to 1.92 and 106.53 to 2.96 g_water_ kg^−1^ h^−1^, when the drying period increased from 1 to 10.5, 1 to 8.0 and 1 to 6.0 h, respectively, for 50, 60 and 70 °C drying temperature for hybrid solar drying system. For oven drying system, the drying rate of marjoram leaves significantly decreased from 53.98 to 0.171, 63.74 to 1.25 and 112.31 to 1.59 g_water_ kg^−1^ h^−1^, when the drying period increased from 1 to 10.5, 1 to 7.5 and 1 to 4.0 h, respectively, for 50, 60 and 70 °C drying temperature compared to decreased from 90.49 to 2.34%, when the drying period increased from 1 to 13 h, respectively, for 3 cm layer thickness for solar drying system.

For hybrid solar and oven drying systems, the results show that the drying rate of marjoram leaves decreases as drying temperature rises. However, for hybrid solar drying system, the drying rate of marjoram leaves increased from 135.7to 204.6, 82.95 to 156.75, and 51.04 to 106.53 g_water_ kg^−1^ h^−1^, respectively, when the drying temperature increased from 50 to 70 °C for 1, 2 and 3 cm layer thickness. Additionally, the drying rate of marjoram leaves rose when the oven drying temperature was raised from 50 to 70 °C for layers thickness of 1, 2, and 3 cm, respectively, from 130.36 to 223.73, 74.6 to 154.21, and 53.98 to 112.31 g_water_ kg^−1^ h^−1^. These results agreed with those obtained by El-Kashoty et al.^4^, Abd El-Haq et al.^6^ and Amer et al.^37^.

### Energy consumption

**Figure 9:**
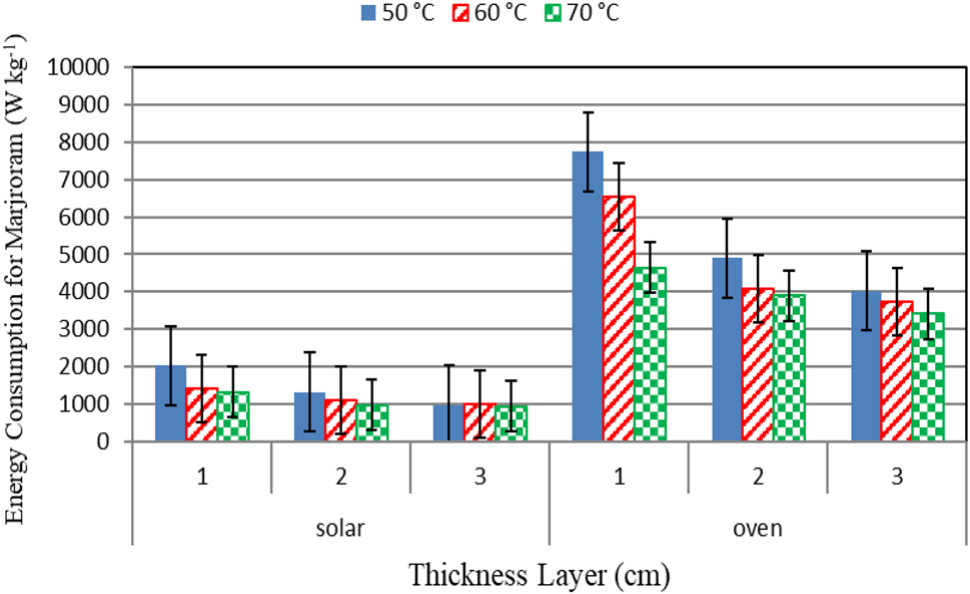
Energy consumption for drying marjoram leaves under different drying systems and different drying temperatures.

Figure 9 shows the energy consumption for drying marjoram under different drying systems (hybrid solar and oven drying) and under different drying temperatures (50, 60 and 70 °C) and different layers thicknesses (1, 2 and 3 cm) at the end of experiment. The results indicate that the highest energy consumption was at temperature of 50 °C and a product thickness of 1 cm was 2017.7 W kg^−1^, and the lowest energy consumption was at a temperature of 60 °C with a thickness of 3 cm and it was 933.3 W kg^−1^ in the hybrid solar system. As for ovens, the highest energy consumption was at a temperature of 50 °C and a thickness of 1 cm and it was 7737.7 W kg^−1^, while the lowest energy consumption at 70 °C and 3 cm thick was 3408.8 W kg^−1^.

The results also indicate that, the energy consumption for drying marjoram under oven drying system was more than those of hybrid solar drying system. It could be seen that, the average energy consumption for drying basil was 1430.4, 1070.3 and 1164.4 and 5552.8, 4784.9 and 3982.9 W kg^−1^ at 50, 60 and 70 °C drying temperature, respectively, for hybrid solar and oven drying systems. Also, the average energy consumption for drying marjoram was 1579.2, 1122 and 963.9 and 6302.2, 4294 and 3724.4 W kg^−1^ at 1, 2 and 3 cm layer thickness, respectively, for hybrid solar and oven drying systems. The trend of these results agreed with those obtained by Motevali and Minael^9^ and Seyfi et al.^32^.

### Essential oil content of marjoram

**Figure 10:**
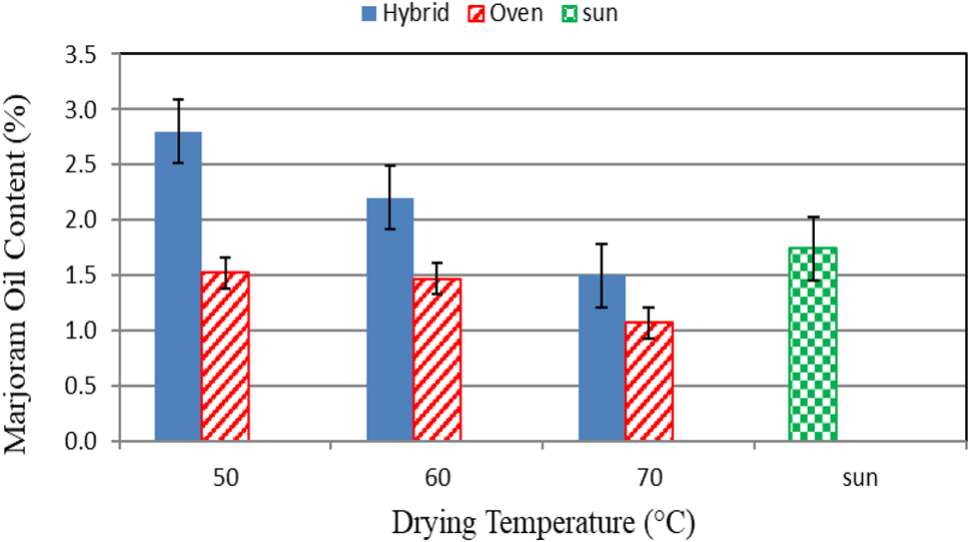
The essential oil content of marjoram leaves that dried in different drying systems and different drying temperatures.

Figure 10 shows the essential oil content of marjoram leaves that dried in different drying systems (hybrid solar and oven drying) and under different drying temperatures (50, 60 and 70 °C) at the end of experiment. The results indicate that the marjoram essential oil content decreases with increasing drying temperature for hybrid solar and oven drying systems, it could be seen that the marjoram essential oil content decreases from 2.80 to 2.10 and 1.52 to 1.07%, when the drying temperature increased from 50 to 70 °C for hybrid solar and oven drying systems, respectively compared to 2.91% for sun drying system. The results indicate that the highest value of the marjoram oil content (2.80%) was obtained when the marjoram dried by hybrid solar drying system at 50 °C. Meanwhile, the lowest value of the marjoram oil content (1.07%) was found at the oven drying system at 70 °C. The trend of these results agreed with those obtained by Abd EL-All et al.^36^.

### Oil compounds of marjoram:

Table 2 shows the oil compound of marjoram leaves that dried in different drying systems (hybrid solar, oven and sun drying) under different drying temperatures (50, 60 and 70 °C) at the end of the experimental period. The results indicate that, the oil compound of marjoram leaves that dried under sun drying system was more than those of hybrid solar and oven drying system. Also, the results indicate that, the oil compound of marjoram leaves decreases with increasing drying temperature. It could be seen that, the α-pinene values were 1.19, 1.07 and 1.07 and 0.95, 0.91 and 0.82% for 50, 60 and 70 °C drying temperature, respectively, for hybrid solar and oven drying systems compared to 0.74and 1.23% for fresh and solar drying system, respectively.

**Table 2:** The oil compound of marjoram leaves that dried in different drying systems and different drying temperatures.

Components	Marjoram Oil Concentration, %							
	Fresh	Solar			Oven			Sun
		Temperature, °C			Temperature, °C			
		50	60	70	50	60	70	
α-Pinene	0.74^a^	1.19^f^	1.07^e^	1.07^e^	0.95^d^	0.91^c^	0.82^b^	1.23^g^
Camphene	1.32^e^	1.32^e^	1.20^d^	1.06^b^	1.21^d^	1.11^c^	0.99^a^	1.31^e^
β-Phellandrene	7.86^e^	8.77^g^	8.27^f^	7.47^d^	6.54^c^	6.15^b^	5.66^a^	8.81^g^
β-Pinene	0.74^d^	0.77^e^	0.69^c^	0.62^b^	0.68^c^	0.63^b^	0.57^a^	0.74^d^
β-Myrcene	2.34^f^	2.34^f^	2.16^e^	1.73^b^	1.92^d^	1.86^c^	1.31^a^	2.18^e^
D-Limonene	8.21^a^	11.46^c^	11.09^c^	11.53^c^	9.49^ab^	9.08^a^	8.69^a^	11.04^c^
Eucalyptol	3.64^d^	1.38^c^	1.24^b^	1.06^a^	4.02^e^	4.35^f^	4.03^e^	1.36^c^
β-Ocimene	4.21^e^	4.40^f^	4.08^d^	3.47^b^	4.04^d^	3.94^c^	2.93^a^	4.47^f^
γ-Terpinene	11.53^a^	15.14^d^	14.75^c^	15.08^cd^	13.23^b^	12.58^ab^	11.98^a^	14.93^c^
Linalool	4.28	1.20^a^	2.55^d^	1.82^b^	2.04^c^	2.63^d^	4.41^e^	1.71^b^
Camphor	4.22^a^	4.83^d^	4.65^b^	4.68^bc^	4.82^d^	4.63^b^	4.56^b^	4.76^d^
Terpinen-4-ol	10.85^e^	3.03^a^	5.82^bc^	4.25^b^	6.13^c^	5.64^b^	8.97^d^	3.46^a^
α-Terpineol	2.79^b^	2.65^a^	2.66^a^	2.69^a^	2.82^b^	3.05^c^	3.14^d^	2.84^b^
Methyl cinnamate	1.74^a^	1.81^a^	1.76^a^	1.89^b^	2.02^cd^	1.98^c^	1.97^c^	1.92^b^
Eugenol	0.84^a^	–	–	–	0.88^a^	0.95^a^	0.87^a^	–
trans-Methyl cinnamate	23.47^a^	28.65^d^	27.44^c^	30.55^f^	26.00^b^	26.13^b^	23.71^a^	29.50^e^
β-Caryophyllene	5.27^a^	5.85^a^	5.70^a^	5.95^a^	5.29^a^	5.00^a^	4.84^a^	6.02^a^
Germacrene D	0.73^a^	0.61^a^	0.65^a^	0.65^a^	0.70^a^	0.95^a^	1.04^a^	0.60^a^
β-Cyclogermacrane	0.99^a^	0.97^a^	0.99^a^	1.08^a^	1.11^a^	1.05^a^	1.11^a^	0.96^a^
γ-Cadinene	2.22^c^	0.97^b^	1.01^b^	0.81^a^	2.74^e^	2.50^d^	2.67^e^	0.76^a^
tau.-Cadinol	2.02^c^	0.86^b^	0.91^b^	0.72^a^	2.54^e^	2.12^d^	2.61^f^	0.72^a^
Sabinene	–	0.44^a^	–	0.48^a^	–	–	–	–
α-Terpinene	–	0.79^a^	0.70^a^	0.72^a^	–	0.53^a^	0.59^a^	0.66^a^
p-Cymene	–	0.57^a^	0.63^a^	0.64^a^	0.68^a^	0.61^a^	0.84^a^	–
Terpinolene	–	–	–	–	–	0.71^a^	0.86^a^	–
γ-Elemene	–	–	–	–	0.57^a^	0.59^a^	0.73^a^	–
Spathulenol	0.74^a^	1.19^a^	1.07^a^	1.07^a^	0.95^a^	0.82^a^	0.91^a^	1.03^a^
Caryophyllene oxide	1.32^a^	1.32^a^	1.20^a^	1.06^a^	1.21^a^	1.11^a^	0.99^a^	1.29^a^
epi-Cubenol	7.86^c^	8.77^e^	8.27^d^	7.47^c^	6.15^b^	6.54^b^	5.66^a^	8.79^e^
Shyobunol	0.74^a^	0.77^a^	0.69^a^	0.62^a^	0.68^a^	0.63^a^	0.57^a^	0.74^a^
trans-β-Ionone	2.34^d^	2.34^d^	2.16^c^	1.73^b^	1.92^b^	1.86^b^	1.31^a^	2.32^d^

Means on the same row with different superscripts are significantly different (p < 0.05).

The camphene values were 1.32, 1.20 and 1.06 and 1.21, 1.11 and 0.99% for 50, 60 and 70 °C drying temperature, respectively, for hybrid solar and oven drying systems compared to 1.32 and 1.31% for fresh and solar drying system, respectively. The D-Limonene values were 11.46, 11.09 and 11.53 and 9.49, 9.08 and 8.69% for 50, 60 and 70 °C drying temperature, respectively, for hybrid solar and oven drying systems compared to 8.21 and 11.04% for fresh and sun drying system, respectively. The trend of these results agreed with those obtained by Amer et al.^37^ and Abd El-Haq et al.^38^ whose found that the volatile composition of product decreases with increasing the drying temperature.

### Total costs

**Figure 11:**
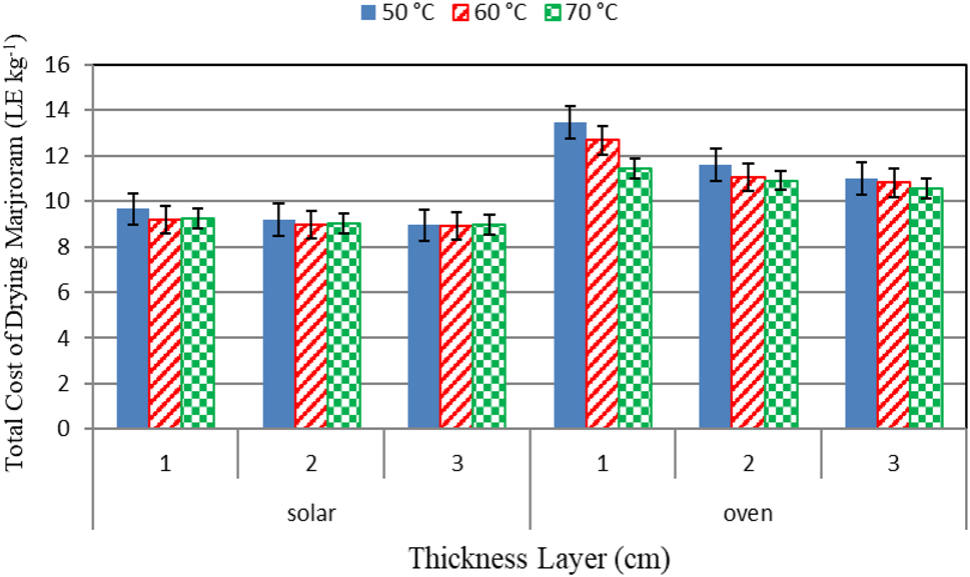
Total cost for drying marjoram leaves under different drying systems and different drying temperatures.

Figure 11 shows the total cost for the marjoram leaves that dried under different drying systems (hybrid solar, oven and solar drying) under different drying temperatures (50, 60 and 70 °C) at the end of the drying period. The results indicate that, the cost of dried marjoram that dried under hybrid solar drying system was less than those of oven drying system. The results indicate that the highest cost of drying marjoram was at a temperature of 50 °C and a thickness of the plant layers 1 cm was 9.65 LE kg^−1^, and the lowest cost consumption was at 60 °C with a thickness of 3 cm, and it was 8.92 LE kg^−1^ in the hybrid solar system. As for ovens, the highest cost was at a temperature of 50 °C and a thickness of 1 cm, and it was 13.48 LE kg^−1^ and the lowest cost consumption was at 70 °C with a thickness of 3 cm, and it was 10.58 LE kg^−1^. The trend of these results agreed with those obtained by Onyenwigwe et al.^39^.

## Conclusion

The marjoram leaves were dried under different conditions with different layer thicknesses. From the results of this study, it is concluded that the average weight losses of marjoram was 74.00% for all treatments under study. The oil content was as high of 2.91% for the product dried under sun drying system, and as low of 1.07% for the product dried under oven drying system. The energy consumption values were 0, 2017.7 and 7737.7 W kg^−1^ for sun, hybrid solar and oven systems, respectively for low layer thickness. Oven drying system recorded 13.48 LE kg^−1^ for oven drying system. The study recommends further studied should be done on increasing alternative the solar drying efficiency and using a renewable energy for drying such as biogas.

## Data Availability

The datasets used and/or analyzed during the current study available from the corresponding author on reasonable request.
